# Selection and identification of a novel ssDNA aptamer targeting human skeletal muscle

**DOI:** 10.1016/j.bioactmat.2022.05.016

**Published:** 2022-05-27

**Authors:** Shuming Sun, Han Liu, Yan Hu, Yanpeng Wang, Mingri Zhao, Yijun Yuan, Yafei Han, Yingying Jing, Jin Cui, Xiaoxiang Ren, Xiao Chen, Jiacan Su

**Affiliations:** a Molecular Biology Research Center, Center for Medical Genetics, Hunan Province Key Laboratory of Basic and Applied Hematology, School of Life Sciences, Central South University, Changsha, 410078, China; b Institute of Translational Medicine, Shanghai University, Shanghai, 200444, China; c Center for Medical Genetics & Hunan Key Laboratory of Medical Genetics, School of Life Sciences, Central South University, Changsha, Hunan, 410013, China; d Molecular Science and Biomedicine Laboratory (MBL), State Key Laboratory of Chemo/Biosensing and Chemometrics, College of Biology, College of Chemistry and Chemical Engineering, Aptamer Engineering Center of Hunan Province, Hunan University, Changsha, Hunan, 410082, China; e Department of Orthopedics Trauma, Shanghai Changhai Hospital, Naval Medical University, Shanghai, 200433, China

**Keywords:** Aptamer, SELEX, Human skeletal muscle, Target delivery, Nanoliposomes

## Abstract

Skeletal muscle disorders have posed great threats to health. Selective delivery of drugs and oligonucleotides to skeletal muscle is challenging. Aptamers can improve targeting efficacy. In this study, for the first time, the human skeletal muscle-specific ssDNA aptamers (HSM01, etc.) were selected and identified with Systematic Evolution of Ligands by Exponential Enrichment (SELEX). The HSM01 ssDNA aptamer preferentially interacted with human skeletal muscle cells *in vitro*. The *in vivo* study using tree shrews showed that the HSM01 ssDNA aptamer specifically targeted human skeletal muscle cells. Furthermore, the ability of HSM01 ssDNA aptamer to target skeletal muscle cells was not affected by the formation of a disulfide bond with nanoliposomes *in vitro* or *in vivo*, suggesting a potential new approach for targeted drug delivery to skeletal muscles via liposomes. Therefore, this newly identified ssDNA aptamer and nanoliposome modification could be used for the treatment of human skeletal muscle diseases.

## Highlights

• Using SELEX, our study selected and identified a human skeletal muscle-specific ssDNA aptamer.
• HSM01 ssDNA aptamer preferentially interacts with human skeletal muscle cells *in vitro* and *in vivo.*
• When linked with nanoliposomes, the skeletal muscle cells targeting ability still remains the same*.*

## Graphical abstract

**Figure:**
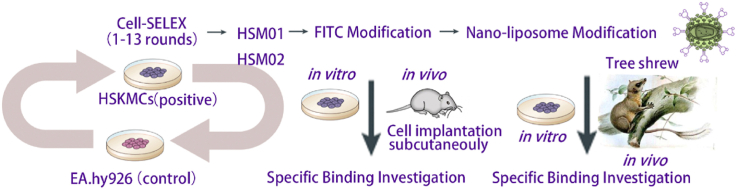

## 1 Introduction

Skeletal muscles account for 30–50% of the bodyweight responsible for body movements and skeletal muscle cells contain up to 50–75% of the proteins in the human body [1]. Skeletal muscle diseases including muscular dystrophy, fibromyalgia, and cerebral palsy et al. remain unsolved and pose great threats to human health [2,3]. Besides, the number of people with degenerative musculoskeletal diseases like sarcopenia is rapidly increasing due to aging population [[4], [5], [6], [7]]. Musculoskeletal diseases are characterized by pain and limitation of general function, severely limiting mobility and flexibility [[8], [9], [10], [11]].

In recent years, drugs have been developed for skeletal muscle diseases such as insulin-like growth factor-1, oligonucleotides, and so on [12,13]. Among them, antisense oligonucleotides (AONs) that bind to the sense target sequence in Duchenne muscular dystrophy have been developed to restore the disrupted reading frame of the dystrophin pre-mRNA [14]. The AONs 2′-*O*-methyl phosphorothioate (2′-OMe PS) (drisapersen) and phosphorodiamidate morpholino (PMO) (eteplirsen) have been tested in clinical trials for exon 51 skipping [[15], [16], [17], [18]]. The US Food and Drug Administration (FDA) approved AONs for clinical use in 2016 [19]. Nevertheless, lacking muscle cell specificity leads to unpredictable systemic adverse effects [20,21]. Thus, targeting strategies are needed to be explored to improve drug efficacy and reduce side effects, and how to efficiently deliver drugs to skeletal muscles is still very challenging.

Aptamers are short (15–100 nt) artificial, single-stranded oligo(deoxy) nucleotides (ssDNA or RNA) with unique three-dimensional (3D) conformations that can form stable complexes with target molecules [[22], [23], [24], [25], [26], [27], [28]]. Aptamers are selected through an *in vitro* molecular selection method named Systematic Evolution of Ligands by Exponential Enrichment (SELEX). They are oligonucleotide probes that recognize and effectively bind to targets, which provides a promising way for targeted drug delivery to skeletal muscles.

Aptamers have several advantages, including a diverse range of targets (from cells to metal ions), simple synthesis and modification, low toxicity and immunogenicity, small size (approximately 1–2 nm in diameter), and good stability under various environmental conditions [[29], [30], [31], [32], [33]]. In addition, aptamers are synthesized *in vitro,* which reduces contamination by bacteria or viruses [34]. Self-functional aptamers bind to key functional molecules, regulate cell signaling pathways, inhibit cell growth, and induce protein degradation [35]. Non-self-functional aptamers have been used in various biomedical applications, including bio-imaging, *in vitro* and *in vivo* molecular diagnosis, and targeted drug delivery [36].

In the present study, we aimed to select and identify a new aptamer targeting human skeletal muscle cells using the Cell-SELEX. We linked the aptamer to nanoliposomes and evaluated its targeting ability both *in vitro* and *in vivo*.

## 2 Results

### 2.1 Preparation for *in vitro* selection of ssDNA aptamers interacting with human skeletal muscle cells

**Fig. 1:**
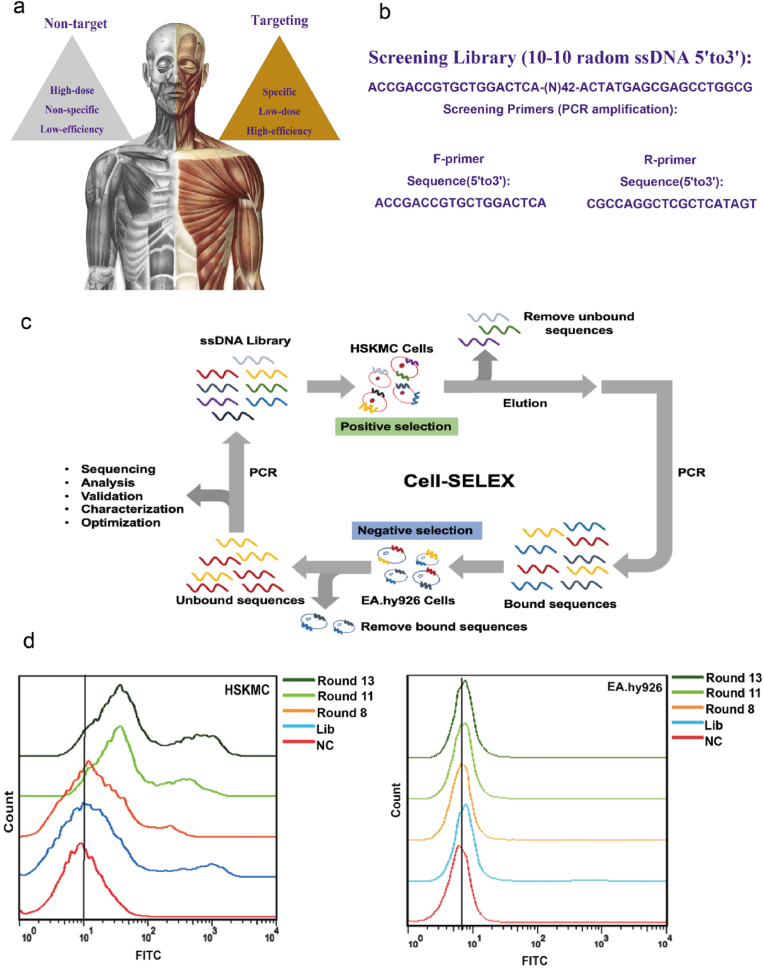
Aptamer Selection Strategy and the Selection. a. Schematic illustration of the target advantages compared to non-target delivery. b. An initial single-stranded DNA library was synthesized, with a 42-nt-long random region in the middle designed and two fixed known primer binding sites on the left and right sides. c. Schematic illustration of the cell-SELEX workflow. d. The flow cytometry experiments of HSKMC and EA. Hy926 with the amplified FITC tagged products after the 0, 8th, 11th, and 13th rounds of screening.

To achieve the goal of the human skeletal muscle cells targeting (Fig. 1a), this study focuses on novel aptamers selection to target human skeletal muscle cells. Firstly, an ssDNA library (Lib) was synthesized, with a 42-nt-long random region in the middle and two fixed known primer binding sites on the left and right sides (Fig. 1b). The 5′-modified primers were synthesized for each selection round (polymerase chain reaction (PCR) amplification). Each selection round was designed in Supplementary Table 1. For each selection round (Supplementary Table 1), the pool of random ssDNA sequences was selected against (positive selection) the target cell line, i.e., human skeletal muscle cells (HSKMCs). In this study, the HSKMC was selected as the positive cell line, while EA. hy926 cells (human umbilical vein endothelial cells) were the negative cell line (Fig. 1c).

### 2.2 Aptamer enrichment assessment by cell-SELEX

Supplementary Table 1 shows the 13 screening rounds of the SELEX process, with HSKMCs being positive cells and EA. hy926 cells as negative control cells. The FITC-labeled initial aptamer Lib (round 0) and amplified FITC-labeled products after the 8th, 11th, and 13th rounds of screening were used for binding experiments with HSKMCs and EA. hy926 cells (Fig. 1d). Flow cytometry was used to examine the binding ability of the fluorescent amplification products to HSKMCs and EA. hy926 cells. As shown in Fig. 1d, the curves for HSKMCs shifted from left to right, while the binding curve for EA. hy926 cells did not shift significantly, suggesting that the specificity of the aptamer was significantly enhanced with an increasing number of screening rounds. By the 11th and 13th rounds, the specificity remained stable, and the amplified products could be used for DNA sequencing.

### 2.3 Identification of human skeletal muscle cell-specific ssDNA aptamers

**Fig. 2:**
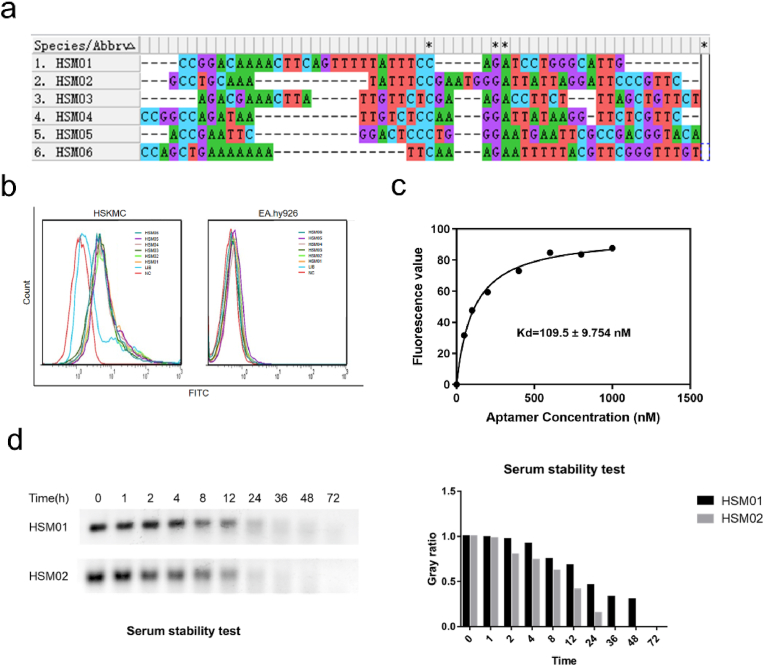
Characteristics of DNA aptamers. a. Alignment of Top 6 sequencing results from HSM01-06. b. Flow cytometry results of synthesized HSM01-06 binding to HSKMC and EA. Hy926 cells. c. Binding dynamic curve and statistical results for HSM01. d. The serum stability of HSM01 and HSM02 in 10% serum incubation medium. *p < 0.05.

High-throughput sequencing was performed on the amplification products of the 11th and 13th rounds, and the results were sorted by sequence read numbers (Fig. 2Aa and Supplementary Table 2). The top six DNA sequences in the 13th round were also included in the 11th round. The top six aptamers in the 13th round of sequencing were selected for *in vitro* synthesis and binding verification experiments (HSM01, HSM02, HSM03, HSM04, HSM05, and HSM06). The 6 aptamers (HSM01, HSM02, HSM03, HSM04, HSM05, and HSM06) were firstly aligned using mega software. The conserved domain shifted from one to another. As shown in Fig. 2b, the 6 aptamers (HSM01, HSM02, HSM03, HSM04, HSM05, and HSM06) and initial screening Lib were tested for binding specificity. The six synthesized aptamers showed good binding specificity, with no significant difference in their binding offset ranges. However, the screening Lib did not show binding specificity.

The top two read numbers in the sequencing report (HSM01 and HSM02) were selected for subsequent experiments. Thereafter, HSM01 and HSM02 were synthesized for the verification experiments. The binding dynamic curve for HSM01 showed that the Kd for binding activity was 109.5 nM (Fig. 2c). The concentration of HSM01 used was 100 nM. The half-life of these aptamers in a 10% serum incubation medium was approximately 8 h (Fig. 2d). The HSM01 and HSM02 ssDNA aptamer was stable in 4–8 h.

### 2.4 HSM01 and HSM02 specifically bind to HSKMCs

**Fig. 3:**
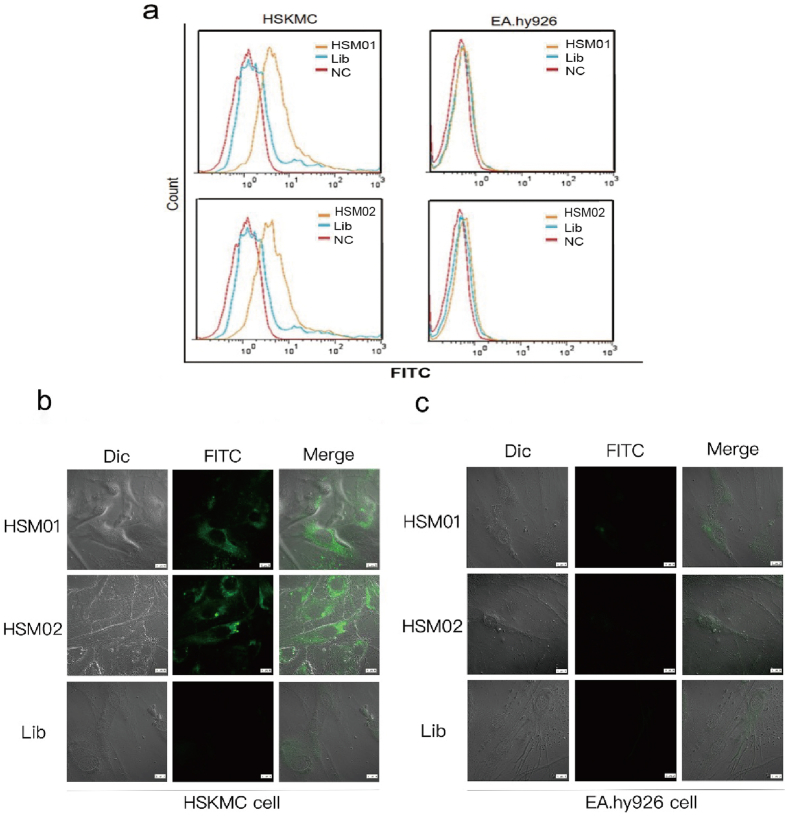
Identification of the DNA aptamer that specifically binds to HSKMC cells. a. HSM01 and HSM02 show curves shift when binding to HSKMC cells and EA. Hy926 cells through flow cytometry assay comparing to NC and Lib control groups, respectively. b. HSM01 and HSM02 show specific fluorescence binding to HSKMC cells through immunofluorescence assay compared to the Lib control group. c. HSM01 and HSM02 show negative fluorescence when incubating with EA. Hy926 cells through immunofluorescence assay compared to Lib control group. Scale bars represent 10 μm.

As shown in Fig. 3a, flow cytometry revealed that HSM01 and HSM02 had better binding ability than the control Lib sequences. We incubated the nucleic acid aptamers HSM01 and HSM02 coupled with FITC, and the Lib coupled with FITC, with the adherent HSKMCs and control EA. hy926 cells. Then, we evaluated the cell localization under confocal microscopy, which showed that the HSM01 and HSM02 aptamers were specifically bound to the cell membrane, and cell plasma, while Lib was weakly bound and not visible (Fig. 3b). The right panel of Fig. 3c showed that the aptamers did not bind to the control EA. hy926 cells and no fluorescence signal was observed.

### 2.5 *In vivo* human skeletal muscle-targeting investigation

**Fig. 4:**
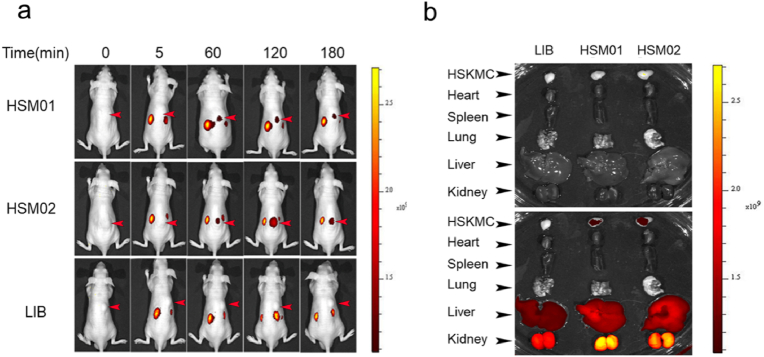
Aptamers *in vivo* targeting experiment. a. Live imaging 5, 60, 120, and 180 min after injection of 3 groups including HSM01 group, HSM02 group, and Lib group. b. Imaging of the heart, lung, spleen, liver, and kidney at 180 min in both white light field and fluorescence field images.

HSKMCs were subcutaneously injected into mice at a dose of 10^7^ cells/mouse. After 48 h, HSM01, HSM02, and the Lib coupled with Cy5.5 fluorophore were injected into the blood vessels of the tail vein of the mice. Using a live animal imaging device and Cy5.5 fluorescence imaging channel (Fig. 4a), at 5 min, 60 min, 120min, and 180 min, images were taken. The images showed that Cy5.5 fluorescence of HSM01 and HSM02 were clearly visualized in the HSKMCs area (marked by a red arrow), whereas Lib was not detectable. At 120 min after injection, the Cy5.5 fluorescence of HSM01 and HSM02 was the strongest and weakened at 180 min. The aptamers were predominantly distributed in high-metabolism organs, such as kidneys and liver in both experimental and control groups (Fig. 4a and b).

To determine the location of the aptamers in mouse organs, we dissected the mice 180 min after the injection and obtained white light field (upper Fig. 4b) and Cy5.5 fluorescence (down Fig. 4b) images of HSKMCs tissues, heart, lungs, spleen, liver, and kidneys. The analysis revealed that HSM01 and HSM02 were localized in the HSKMCs tissues 180 min after the injection, indicating that the aptamer had stable retention in HSKMCs in mice 180 min after a single one-time injection.

### 2.6 The primary analysis of potential protein target for aptamer HSM01

**Fig. 5:**
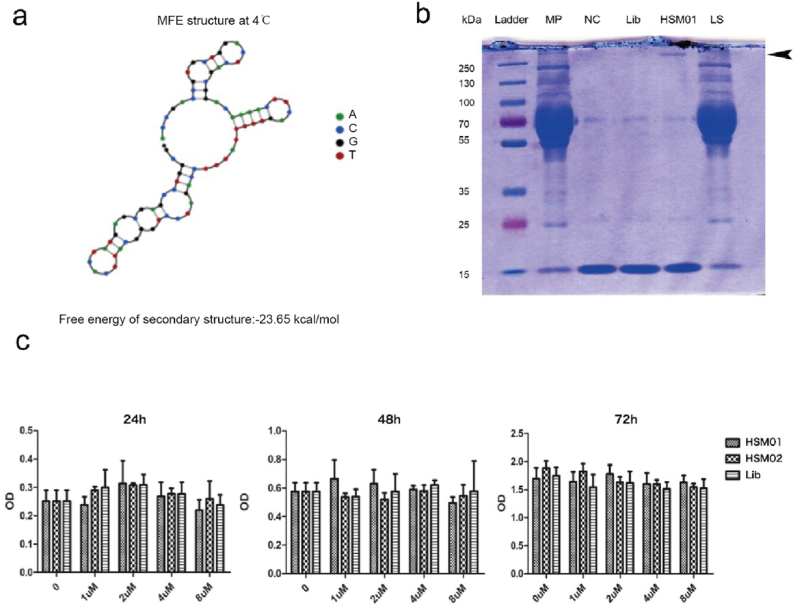
Aptamer HSM01 structure prediction, protein precipitation, and cell treatment experiments. a. Nupack software (http://www.nupack.org/) was used to predict the secondary structure (2D structure) of aptamers. b. the HSKMC membrane protein was incubated with biotin-conjugated HSM01, Lib, or blank, respectively, and then the biotin affinity magnetic beads were added to elute the target protein complex. Then, the HSM01 sample, Lib sample, and NC sample were collected. The remaining supernatant of the HSM01 sample was the last supernatant (LS sample). The above samples (MP sample, HSM01 sample, Lib sample, NC sample, and LS sample) were analyzed through SDS-PAGE electrophoresis and Coomassie Brilliant Blue Staining. A very specific protein band in the HSM01 lane was found above 250 kd. The band and control were sent for LC-MS/MS QSTAR analysis (Supplementary Table 3 and 4). c. The treatment of HSM01 and HSM02 on HSKMCs showed the effect of the aptamer on cell proliferation in 24 h, 48 h, and 72 h, respectively.

Firstly, the Nupack software was used to predict its 2D structure (Supplementary Table 5). A stem-loop structure of HSM01 formed (Fig. 5a). This structure was the potential interaction basis. Secondly, biotin-conjugated HSM01 and biotin-conjugated Lib were synthesized for precipitation. As shown in Fig. 5b, the membrane protein (MP) sample of HSKMCs was collected. The MP was incubated with biotin-conjugated HSM01, Lib, or blank samples, and the biotin Affinity magnetic beads were added to precipitate the biotin-conjugated HSM01 or Lib protein complex. Then, the HSM01, Lib, and NC samples were collected. The remaining supernatant of HSM01 was considered the last supernatant (LS). The samples (MP, HSM01, Lib, NC, and LS) were analyzed using SDS-PAGE and Coomassie Brilliant Blue staining. Interestingly, a specific protein band in the HSM01 lane was found above 250 kd (Fig. 5b). The band and control were sent for LC-MS/MS analysis (QSTAR; Applied Biosystems, Waltham, MA, USA) (Supplementary Tables 3 and 4). The mass spectrometry results showed that fibronectin was the most abundant protein in the HSM01 sample, followed by collagen-3 and the filament protein actin. For further research, a more experimental design will be conducted to verify the target of HSM01. Thirdly, we did co-culture of the HSM01 and HSKMCs to make sure there are no fatal effects of HSM01 on HSKMCs. In Fig. 5c, the treatment of HSKMCs with HSM01 or HSM02 for 24, 48, and 72 h showed no significant effects on its cell proliferation (Fig. 5c).

### 2.7 Nanoliposome-linked ssDNA aptamer design and characterization

**Fig. 6:**
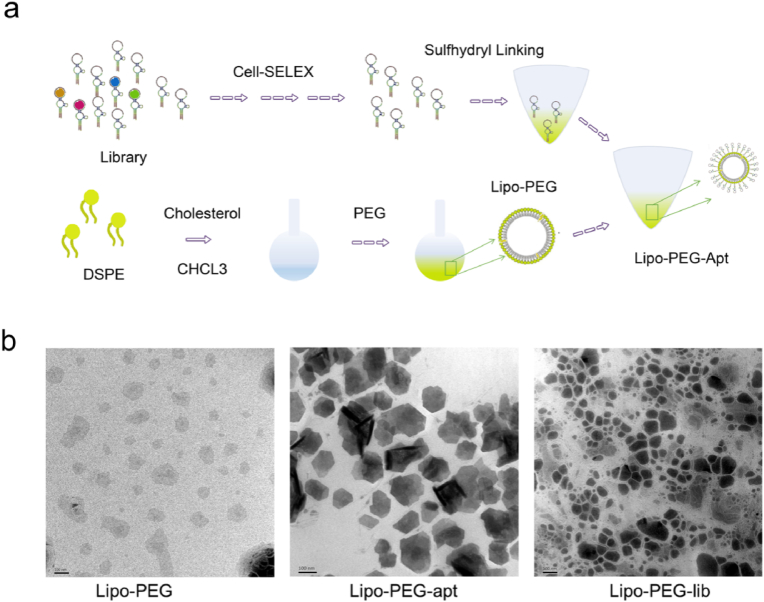
Design and synthesis of Lipo-PEG-apt. a. Schematic illustration of Lipo-PEG-apt synthetization. The reverse evaporation method was used to encapsulate nanoliposomes. DSPE: Distearoyl Phosphoethanolamine; PEG: Polyethylene glycol; Lipo: liposome. b. Transmission electron microscopy showed the synthesized products. The diameter of the HSM01-liposome complex was concentrated at around 100 nm. Scale bars represent 100 nm.

To enhance the function of aptamers, we designed nanoliposomes linked to HSM01 for the targeted transport of drugs and oligonucleotides. Following the schematic diagram in Fig. 6a, reverse evaporation was used to encapsulate nanoliposomes. The designed nanoparticles had a diameter of around 100 nm (Fig. 6b). To couple the nanoparticles to the nucleic acid aptamer with specific targeting ability, a sulfhydryl group (-SH) was added to the HSM01 3′ tail. In addition, polyethylene glycol (PEG) was inserted into the liposome nanoparticles to form stable sulfhydryl bonds with the 3’ tail of aptamers. Cholesterol was added to adjust the fluidity and enhance the stability of the nanoparticles. The 5′ end of HSM01 was conjugated with FITC fluorescence to allow detection of the synthesized nanoparticles. Finally, the HSM01-liposome complex (Lipo-PEG-apt) was successfully built.

**Fig. 7:**
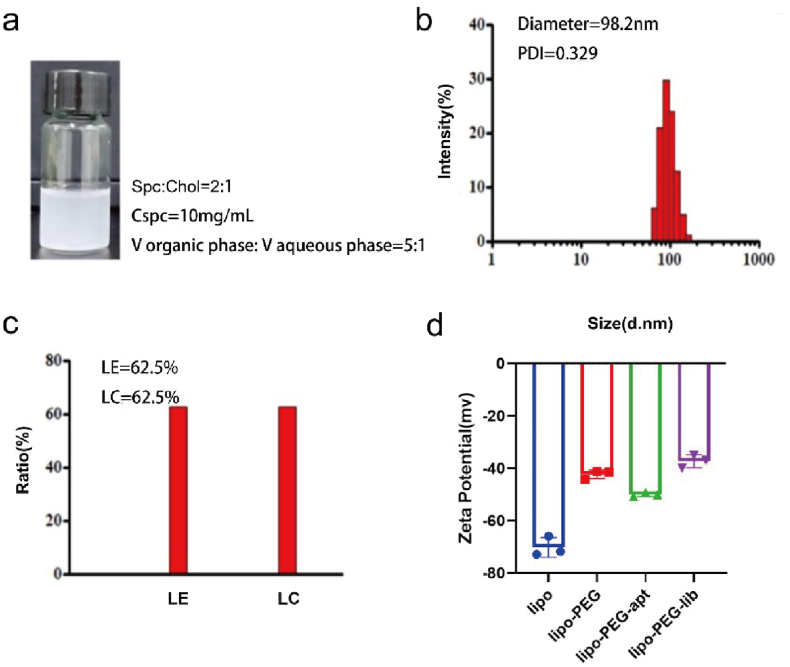
Characteristics of Lipo-PEG-apt. a. Synthesized products' gross appearance and synthesized conditions. b. The test of the diameters. c. The test of the LE (liposome encapsulation rate), and LC (liposome drug-carrying rate). d. Zeta potential test.

The gross appearance of Lipo-PEG-apt was shown (Fig. 7a). The diameter and uniformity of the Lipo-PEG-apt were evaluated and showed a diameter of almost 100 nm and good uniformity (Fig. 7b). The LE (liposome encapsulation rate), and LC (liposome drug-carrying rate) were 62.5% (Fig. 7c). Zeta potential results showed that all four groups presented a negative charge (Fig. 7d).

### 2.8 HSM01-liposome complex targets human skeletal muscle *in vitro*

**Fig. 8:**
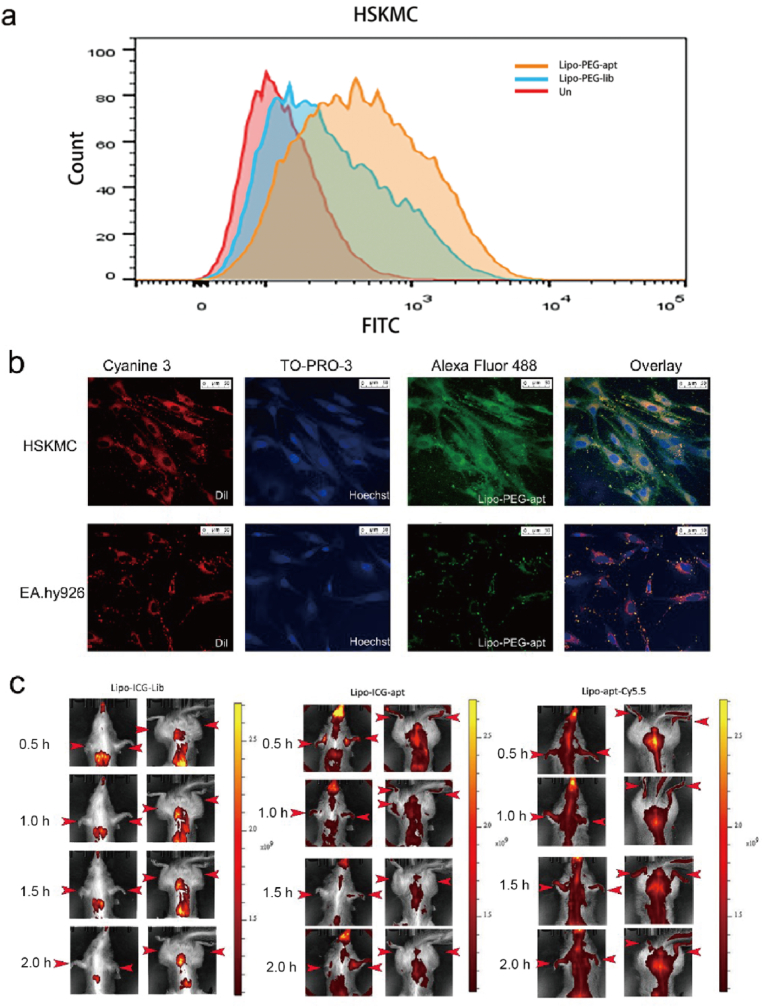
Lipo-PEG-apt targeting specificity assessment *in vitro* and *in vivo*. a. The flow cytometry experiment exhibited HSM01-liposome complex (Lipo-PEG-apt) and Lipo-PEG-Lib binding peaks. b. Immunofluorescence showed green fluorescence of HSM01-liposome complex (Lipo-PEG-apt), red fluorescence (Dil, cell membrane dye), and blue fluorescence (hoechst33342). c. Tree shrew were set in three groups, the control group was liposome conjugated with ICG fluorescence and control nucleic acid substrate (Lib) to form constituted complex (Lipo-ICG-Lib), and a group of liposomes ICG fluorescence and experimental group nucleic acid HSM01 coupling (Lipo-ICG-apt). The third group was liposomes without ICG fluorescence, but HSM01 coupled with the Cy5.5 subunit to form the experimental group (Lipo-apt-Cy5.5). The images represented 0.5 h, 1 h, 1.5 h, and 2 h, respectively. Scale bars represent 50 μm.

To confirm the specific target ability of the aptamer-liposomes, the immunofluorescence and flow cytometry experiments were used to test whether Lipo-PEG-apt can target HSKMCs. As shown in Fig. 8a, Lipo-PEG-apt showed a similar specific binding ability to aptamers. The flow cytometry experiment exhibited a significant peak binding ability of Lipo-PEG-apt than Lipo-PEG-Lib, which confirmed the ability of Lipo-PEG-apt to target HSKMCs. To further confirm the specific binding ability of Lipo-PEG-apt to HSKMCs cells, immunofluorescence experiments were performed. Results showed that the Lipo-PEG-apt was bound to HSKMCs with strong fluorescence intensity. Compared to the control group, the Lipo-PEG-apt was colocalized in the region of red fluorescence (cell membrane dye), which did not overlap with the Hoechst blue fluorescence (Fig. 8b).

### 2.9 Nanoliposome-linked ssDNA aptamer targets skeletal muscle in tree shrews

The nude mice experiments showed the subcutaneously implanted HSKMCs targeted (Fig. 4). From the figures, it showed that the aptamers did not target the mouse skeletal muscle area, especially the mouse limb part. To further verify its target ability, tree shrews were chosen for verification. The tree shrew is an experimental animal developed in China useful for studying nerves, the digestive system, hepatitis A and B, rotavirus, and cancers. Previous studies of tree shrew, including genome sequencing analysis, reported its close relationship with primates (∼93.4% genomic similarity) in terms of tissue anatomy, physiology, biochemistry, the nervous system (brain function), and the metabolic system. Previous studies of proteomics have shown that tree shrew has liver and skeletal muscles closer to those of humans compared to mice and rats.

To determine whether Lipo-PEG-apt has a specific targeting ability *in vivo*, and also to determine whether the targeting ability comes from aptamer but not from liposomes *in vivo*, tree shrew was used and assigned into three groups: a control group (liposomes conjugated with ICG fluorescence and nucleic acid substrate (Lib) to form the Lipo-ICG-Lib complex) and two experimental groups (liposomes conjugated with ICG fluorescence coupled with HSM01 (Lipo-ICG-apt), and liposomes without ICG fluorescence, coupled with Cy5.5 conjugated HSM01 (Lipo-apt-Cy5.5)). The images were obtained at 0.5, 1, 1.5, and 2 h. The Lipo-ICG-Lib group showed no fluorescence in skeletal muscle tissues, especially in the limb part of the tree shrew, though fluorescence was visible in the liver and kidney (Fig. 8c). In contrast, the Lipo-ICG-apt and Lipo-apt-Cy5.5 groups showed strong fluorescence in the limbs and abdominal muscle tissues. In the Lipo-apt-Cy5.5 group, the HSM01 aptamer was tagged with Cy5.5 fluorescence, suggesting that HSM01 could not only target muscles but also guide the nanoliposome complex. Normally, liposomes could not target the limbs and abdominal muscle tissues. Under the guidance of HSM01, the Lipo-ICG-apt group showed target ability similar to the Lipo-apt-Cy5.5 group.

### 2.10 Biosafety assessment of lipo-PEG-apt on rat liver and kidneys

**Fig. 9:**
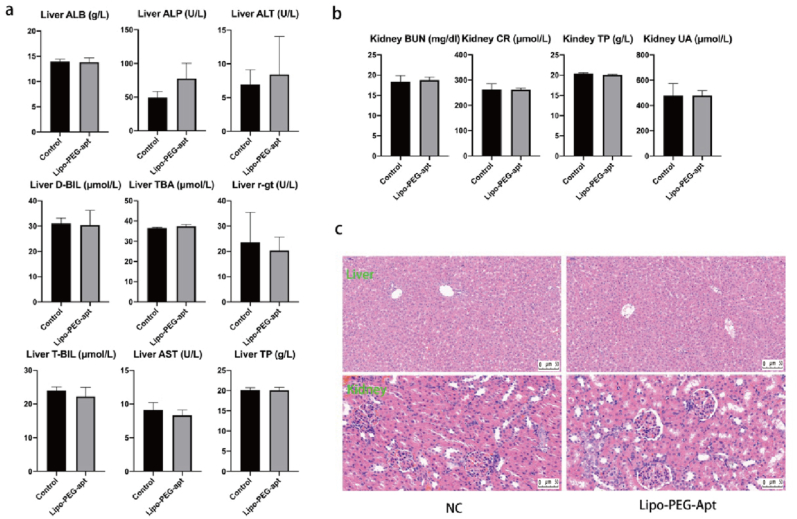
Lipo-PEG-apt metabolic and histologic analysis in rat liver and kidney. a. Levels of ALT (alanine aminotransferase), AST (aspartate aminotransferase), ALB (albumin), ALP (alkaline phosphatase), DBIL (direct bilirubin), TBIL (total bilirubin), r-gt (glutamyl transpeptidase), TBA (total bile acid), TP (total protein) in liver. b. Levels of BUN (urea nitrogen), CR (serum creatinine), UA (uric acid), and TP (total protein) in serum. c. The sections of the liver and kidney were stained with hematoxylin and eosin. Scale bars represented 50 μm.

To assess the biosafety of the synthesized Lipo-PEG-apt, the control group was injected with phosphate-buffered saline (PBS) and experimental rats were injected with Lipo-PEG-apt. In the liver, Lipo-PEG-apt is mainly used to detect the levels of alanine aminotransferase, aspartate aminotransferase, albumin, alkaline phosphatase, direct bilirubin, total bilirubin, glutamyl transpeptidase, total bile acid, and total protein (Fig. 9a). The main indicators of kidney functions are urea nitrogen, creatinine, uric acid, and total protein. The results showed that the levels were not significantly different between the two groups. The liver and kidneys did not reveal significant differences between the groups in histological structure and inflammation (Fig. 9c).

## 3 Discussion

In the present study, we screened and found an ssDNA aptamer that could target HSKMCs both *in vitro* and *in vivo*. Both mouse targeting experiment and tree shrew targeting experiment *in vivo* proved that the ssDNA aptamer could target the muscle cells and muscle tissue. The aptamer-based nanoliposome could also target human skeletal muscles *in vitro* and *in vivo*. These findings could facilitate targeted drug delivery to human skeletal muscle for treatment.

Skeletal muscles attached to bones by tendons, produce force to help us move under voluntary control [[37], [38], [39]]. Skeletal muscle diseases can significantly affect the movement and living quality of patients [[39], [40], [41], [42]]. Currently, various skeletal muscle disorders including muscular dystrophy, cerebral palsy, dermatomyositis, myasthenia gravis, etc. have posed great threats to human health. The incidence of musculoskeletal diseases has significantly increased due to an aging population [[43], [44], [45]].

The current treatment options for most skeletal muscular diseases are oral drugs, such as Everisdy for spinal muscular atrophy, and Prednisone for polymyositis. Although such drugs have shown good potential as therapeutic agents, the bioavailability and toxicity of drugs limit their further application. Notably, targeted delivery of drugs can minimize side effects and increase the local concentration of therapeutics. Therefore, to develop a human skeletal muscle targeted drug delivery strategy is urging.

As RNA/DNA oligonucleotide molecules, aptamers can specifically bind to targeted complementary molecules with potential diagnostic and therapeutic applications [46]. Aptamers bind to the target in a way similar to the lock and key mechanism [47]. Aptamers can offer many therapeutic and diagnostic options for biomedical and pharmaceutical applications due to their advantages of simple *in vitro* selection and production, ease of conjugation and modification, low immunogenicity, and high stability [48]. The aptamer is a promising approach [49] for drug delivery into skeletal muscles [50]. Aptamers targeting various tissues and organs have been reported [[51], [52], [53], [54]]. The cardiac troponin targeted ssDNA aptamer has been developed and applied clinically. Avβ3 protein-targeted ssDNA aptamer could reduce the proliferation and migration of smooth muscle cells [55]. An RNA aptamer could also target smooth muscle cells and inhibit neointimal formation [56]. Among these aptamers, some of them show cell-specific targeting ability and cell function ability, and some only show targeting ability. Nevertheless, currently, there have been no aptamers reported targeting human skeletal muscle cells, though a previous study showed a screened RNA aptamer could target mouse skeletal muscle cells [57].

Currently, there has been no reports on aptamers targeting human skeletal muscles. A study by Philippou et al. [57] reported RNA aptamers targeting mouse skeletal muscles. They showed that the aptamer could bind to mouse C2C12 muscle cells *in vitro*, but it lacked the *in vivo* data. Only cross sections from the tibialis anterior muscle of wild-type mice show the localization. In our study, we showed that the aptamers screened could specifically target human skeletal muscles *in vitro* and *in vivo*.

In our *in vivo* research data, we used nude mice for imaging. HSKMCs were subcutaneously injected into the back of the nude mice. The aptamer screened in our study shows its specific binding ability to HSKMCs but not to mouse skeletal muscles *in vivo*. Besides, the aptamer reported is RNA aptamer. In this case, it is unstable both *in vivo* and *in vitro*. The serum stable test is very important for further application.

The animal model limited the study of aptamers targeting human skeletal muscles. Therefore, in this study, we used tree shrew (*Tupaia belangeri chinensis*). Tree shrews have many advantages including small body size, low cost of feeding and maintenance, and short reproductive cycle and life span. Importantly, they have a more close relationship with primate animals than rats [58]. The genome analysis already showed a close relationship with primate animal models [59]. Further investigation found that the liver and the skeletal muscle of tree shrew are closer to primates than to the mouse and rat, by comparing their proteomic data in the liver and skeletal muscle [60]. Because of these characteristics, infectious viruses (SARS-CoV-2, COVID-19, Hepatitis viruses (HBV and HCV), and so on), cancer, depression, drug addiction, diabetes, and so forth [61] have widely employed this animal model. In this study, we found the aptamer selected could specifically accumulate in the skeletal muscles of the tree shrew compared to mice, which further indicated that the aptamer could target human skeletal muscles.

There are many other methods for aptamer screening including cell-SELEX, atomic force microscopy SELEX (AFM-SELEX), capture-SELEX, immunoprecipitation-coupled SELEX (IP-SELEX), artificially expanded genetic information system-SELEX (AEGIS-SELEX), capillary electrophoresis-SELEX (CE-SELEX) [62]. In this case, we used the reliable and classic cell SELEX technique in aptamer screening [63]. In our subsequent screening design, we used EA. hy926 cells as control cells, compared to the experimental cell HSKMCs. We also used other cell types, such as MDA-MB-231, MDA-MB-468, Cal-27, A549, ARP-1, K562, B cell, and SKM-1 cells, to assure its specific targeting ability to HSKMCs but not to other types of cells.

The biosafety of aptamers has been validated by many studies [64,65]. As shown in this study, various biochemical indexes and staining analyses of the liver and kidney indicated that the aptamer has good biocompatibility. As an ssDNA, aptamer-based therapy belongs to gene therapy, which is a promising therapeutic strategy. However, the limitations of aptamers mainly lie in their stability, production costs, potential biosafety, and off-target possibility. Therefore, *in vivo* experiments are still needed to characterize the clearance rate, toxicity, and other issues of aptamers for clinical application. More research is still needed to improve their stability, reduce their production costs, and build perfect preclinical models to accelerate clinical application. In fact, only one aptamer, Pegaptanib, has been approved by the US Food and Drug Administration (FDA) to date [66]. Pegaptanib is used for the treatment of neovascular (wet) age-related macular degeneration. New aptamers such as ARC1779 [67], AS1411 [68], and BAX499 [69] are being approved for clinical evaluation. To be sure, with the development of large and large-scale synthetic oligonucleotides, aptamers are expected to move faster towards clinical evaluation and eventual FDA approval. In the future, we will see more and more aptamers and aptamer-like drugs in the clinic.

To specifically deliver drugs to skeletal muscle tissues, a nanocarrier should be employed. There are many nano-materials for nano-modification of aptamer. Among them, liposomes are classic, most studied, and widely used in pharmaceutical industries [70]. With its 50–450 nm size range and the analogous to the cell membranes, they are used to be good drug delivery vehicles [71]. PEG conjugated lipids we used in this study already have various FDA proven medicines, such as liposomal doxorubicin in combination with bortezomib in multiple myeloma treatment [72]. In our study, we inserted the aptamer into the liposome with PEG and constructed Lipo-PEG-Apt which showed negative charges with long-term circulation and an average particle diameter of 98.2 nm. The nanocomplex serves as a targeted drug delivery system to human skeletal muscles with a potential clinical translational application.

In conclusion, in this study, we developed an aptamer (HSM01) that targets human skeletal muscles and used it to create aptamer-targeted liposomes. This strategy could be used for the targeted transport of drugs to human skeletal muscles in muscle diseases.

## 4 Materials and methods

### 4.1 Cell culture

Skeletal muscle cells (HSKMCs; PCS-950-010; ATCC, Manassas, VA, USA) and EA. hy926 cells (CRL-2922; ATCC) were purchased from ScienCell Research Laboratories (Carlsbad, CA, USA). HSKMCs were cultured at 37 °C and CO_2_ (5%) in a cell incubator with fetal bovine serum (FBS, 5%), skeletal muscle cell growth supplement (SkMCGS, 1%), and penicillin/streptomycin (P/S, 1%). EA. hy926 cells were cultured in Dulbecco's modified Eagle's medium (DMEM) medium supplemented with FBS (10%) and P/S.

### 4.2 Preparation of the ssDNA library

The ssDNA library and primers were obtained by Sangon Biotechnology Co., Ltd. (Shanghai, China). The initial Lib included a 40 nt randomized region and 20 nt primer sequences with no label. For PCR amplification, the forward primers were labeled with FAM (5′-FAM-ACC GAC CGT GCT GGA CTC A-3′) and the reverse primer was labeled with biotin (5′-biotin-CGC CAG GCT CGC TCA TAG T). The oligonucleotide sequences were synthesized by Sangon Biotechnology Co., Ltd.

### 4.3 Aptamer and control sequences

To obtain confocal images and perform flow cytometry, the aptamer and control sequences were synthesized by Sangon Biotechnology Co., Ltd. Oligonucleotides were received as a lyophilized powder and resuspended in RNase-Free water (Solarbio, Beijing, China) at a stocking concentration (100 mM). Oligonucleotides were modified by the addition of 5′-FAM.

### 4.4 *In vitro* selection of ssDNA aptamer

HSKMCs and EA.hy 926 cells were cultured as follows. The cell-SELEX procedure was adapted from previous studies. The washing buffer contained PBS (0.01 M), glucose (4.5 g/L), and MgCl_2_ (5 mmol/L). Then, yeast tRNA (0.1 mg/mL) and bovine serum albumin (BSA) (1 mg/mL) were added to the washing buffer to prepare the binding buffer. The initial ssDNA Lib was dissolved in RNase-Free water (Solarbio, Beijing, China) at a stocking concentration (100 mM) and then was diluted to 100 nM (working concertation) in cell suspensions or solutions to use. The sequence was treated at 95 °C for 10 min and then chilled on ice for 10 min to create a 3D structure before incubation. After 1 h of incubation with the HSKMCs at 4 °C, a random sequence in the Lib against target cells was bound to the cell surface. The cells were washed twice for 2 min with 2 mL of washing buffer to remove the unbound ssDNA. Cells and bound ssDNA were harvested and denatured at 95 °C for 10 min. The solution was centrifuged to separate the sequences from cell sediment, etc. The solution was then subjected to PCR with the previously described amplification primers. To reduce non-specific amplification by PCR, various annealing temperatures and cycle numbers were tested; 55 °C was the best annealing temperature for PCR. The PCR amplification procedure was as follows: 95 °C for 30 s, 60 °C for 30 s, 72 °C for 30 s (12–16 cycles), and 72 °C for 5 min. To capture the biotinylated, double-stranded DNA, streptavidin-coated Sepharose beads were used. FAM-coupled single-stranded oligonucleotides were isolated from the beads using 0.2 M NaOH. The NAP-5 column was used to desalt the purified PCR products, which were used in the next screening rounds.

To get the selection Lib for the next rounds, please refer to the methods “ssDNA pool incubation and DNA amplification” part. The amplification products could be used as the selection Lib for the subsequent cell-SELEX rounds. In subsequent screening rounds, the selection Lib was incubated with EA. hy926 cells. After incubation, the collected supernatant solution was incubated with HSKMCs for positive selection. The selection conditions were gradually made more stringent from the 1st to 13th round, to enhance the screening efficiency by decreasing the incubation time with HSKMCs (from 1 h to 35 min) and the number of HSKMCs, and increasing the incubation time with EA. hy926 cells (from 10 to 40 min) and a number of EA.hy 926 cells. The final ssDNA Lib of the 13th round was directly sequenced after testing its binding ability.

### 4.5 ssDNA pool incubation and DNA amplification

The aptamer pool was heated, cooled, and naturally folded in 1 mL of binding buffer. The binding buffer was based on Dulbecco's phosphate-buffered saline (D-PBS) supplemented with 4.5 g/L of glucose, 5 mmol/L of MgCl_2_, 0.1 mg/mL of yeast tRNA, and 1 mg/mL of BSA. Then, the prepared buffer (500 μL) was added to the HSKMCs or EA. hy 926 cells centrifuged pellets following the cell-SELEX workflow. After incubation on ice for 45 min, the cells were washed twice with washing buffer to remove the unbound sequences. The washing buffer was based on D-PBS supplemented with 4.5 g/L of glucose and 5 mmol/L of MgCl_2._ The incubated and then washed cells were then treated in a 95 °C water bath for 10 min, then cooled for 10 min in cold water to room temperature. With 5000 rpm, 4 °C, 3 min centrifuging, the supernatant was collected for the next PCR amplification. The PCR amplification system consisted of ribozyme free water (655 μl), 10X reaction buffer (100 μl), dNTP (60 μl), forward primer (15 μl), reverse primer (15 μl), template sequence (150 μl), and Taq enzyme (5 μl). The DNA amplification procedure was 14 cycles at 95 °C for 30 s, 55 °C for 30 s, 72 °C for 30 s, and 72 °C for 5 min.

### 4.6 Sequencing

The PCR products were collected and incubated with streptavidin-coated Sepharose beads. The ssDNA was then isolated, purified. High-throughput sequencing was performed on the amplification products of the 11th and 13th rounds using Illumina MiSeq (Sangon Biotech Co., Ltd. Shanghai, China).

### 4.7 Secondary structure of ssDNA

Nupack software (http://www.nupack.org/) was used to predict the secondary structure of aptamers.

### 4.8 Immunofluorescence and confocal microscopy

HSKMCs were cultured in 24-well plates (3 × 10^4^ cells/well) for 24 h with poly-lysine-coated microscopy square slides placed. Make sure cells were about 70–80% convergence on the coated slides. Then, the cells were incubated for 60 min with 250 μL FAM-labeled aptamers in BSA (1%). Then, the cells were again washed with PBS and stained with 4′, 6-diamino-2-phenylindole for 90 s Hoest33342 was used to stain the nucleus. A confocal microscope (LAS X SP-5; Leica, Germany) was used for obtaining images. Among them, Lipo concentration 10 mg/mL, ICG concentration 0.75 mg/mL, aptamer-FAM concentration 10 μM.

### 4.9 Flow cytometry

The ability of the aptamer for target binding was assessed using flow cytometry. HSKMCs were washed with PBS and suspended in cold binding buffer. In HSKMCs and EA. Hy926 cells, Lib, Apt, Lib-Lipo, Apt-Lipo, Lib-Lipo-Apt, and Apt-Lipo-Apt were fluorescence-labeled at 300 nm using FAM at 4 °C for 45 min. The initial Lib was used as the negative control. Then, the cells were washed with cold binding buffer to remove the unbound ligands and analyzed by flow cytometry. The mean fluorescence intensity of the background was subtracted from the mean fluorescent intensities of the samples. The binding assays were performed three times.

### 4.10 Nanoliposome-linked ssDNA aptamer design

To enhance the function of aptamers, we designed nanoliposomes linked to HSM01 for the targeted transport of drugs and oligonucleotides. Reverse evaporation was used to encapsulate nanoliposomes. The designed nanoparticles had a diameter of around 100 nm. To couple the nanoparticles to the nucleic acid aptamer, a sulfhydryl group (-SH) was added to the HSM01 3′ tail. In addition, polyethylene glycol (PEG) was inserted into the liposome nanoparticles to form stable sulfhydryl bonds with the 3’ tail of aptamers. To adjust the fluidity and enhance the stability of the nanoparticles, cholesterol was added. The 5′ end of HSM01 was conjugated with FITC fluorescence to allow detection of the synthesized nanoparticles. Finally, the HSM01-liposome complex (Lipo-PEG-apt) was successfully created. All liposomes, including Liposomes with PEG inserted (Lipo-PEG), the control library conjugated liposomes (Lipo-PEG-lib), and the aptamer conjugated liposomes (Lipo-PEG-Apt) are synthesized in the methods described above.

### 4.11 Animal experiment design

*In vivo* imaging of nude mice: HSKMCs were cultured, collected, and subcutaneously injected into the right side of the back of nude mice to grow cell clusters. Fluorescent material was injected into the mice through the tail vein. Fluorescence was observed at different positions and time points (0, 30, and 60 min) using a small animal imager.

*In vivo* imaging of tree shrews: Fluorescent materials were injected into the tree shrews through the tail vein, and fluorescence was observed at different positions and time points (0, 30, 60, 90, and 120 min) were observed using a small animal imager. Among them, Lipo concentration 6 mg/mL, ICG concentration 0.45 mg/mL, apt-Cy5.5 concentration 10 μM and Lib-Cy5.5 concentration 10 μM was injected into each rat with 200 μL liposome complex.

Metabolism rat experiments: lipo concentration 6 mg/mL, apt concentration 10 μM and was tail vein injected into each rat with 200 μL Lipo-PEG-apt complex.

### 4.12 Statistical analysis

All experiments were performed three times. GraphPad Prism 7 (GraphPad Software Inc., San Diego, CA, USA) was used to calculate the mean and SD, and graph the results. For paired comparisons between groups, Student's t-test was used. P < 0.05 was considered significant. We selected representative images according to the mean/median value of each group.

## CRediT authorship contribution statement

**Shuming Sun:** Visualization, Data curation, Writing – original draft, Formal analysis, Funding acquisition. **Han Liu:** Investigation, Methodology, Validation. **Yan Hu:** Methodology, Formal analysis. **Yanpeng Wang:** Validation, Formal analysis. **Mingri Zhao:** Data curation, Investigation. **Yijun Yuan:** Data curation, Formal analysis. **Yafei Han:** Investigation, Validation. **Yingying Jing:** Investigation, Validation. **Jin Cui:** Resources, Supervision, Writing – review & editing. **Xiaoxiang Ren:** Resources, Supervision, Writing – review & editing. **Xiao Chen:** Resources, Methodology, Formal analysis, Software. **Jiacan Su:** Conceptualization, Resources, Supervision, Writing – review & editing, Project administration.

## Declaration of competing interest

The authors declare no conflict of interest.
