# Supplementary material for: Selection and identification of a novel ssDNA aptamer targeting human skeletal muscle
Source: Bioact Mater. 2022 May 27;20:166–78. doi: 10.1016/j.bioactmat.2022.05.016 (PMC9157180; doi:10.1016/j.bioactmat.2022.05.016)
Supplement: Multimedia component 1 [file mmc1.docx]

**Table 1.Selection conditions**

|  | | | | | | |
| --- | --- | --- | --- | --- | --- | --- |
|  |  | positive selection | | counter selection | |  |
| rounds | ssDNA(pmol) | dimension of culture dish(mm) | incubation time(min) | dimension of culture dish(mm) | incubation time(min) | PCR cycle number |
| 1 | 6800 | 100 | 120 |  |  | 10 |
| 2 | 200 | 100 | 60 |  |  | 16 |
| 3 | 180 | 100 | 60 | 60 | 10 | 12 |
| 4 | 140 | 100 | 60 | 60 | 10 | 16 |
| 5 | 80 | 60 | 60 | 60 | 10 | 16 |
| 6 | 120 | 100 | 60 | 60 | 15 | 14 |
| 7 | 80 | 100 | 60 | 60 | 15 | 12 |
| 8 | 80 | 100 | 45 | 60 | 25 | 12 |
| 9 | 80 | 100 | 40 | 60 | 25 | 14 |
| 10 | 50 | 100 | 35 | 60 | 30 | 14 |
| 11 | 50 | 60 | 35 | 100 | 30 | 16 |
| 12 | 50 | 60 | 35 | 100 | 35 | 16 |
| 13 | 40 | 60 | 35 | 100 | 40 | 15 |
|  |  |  |  |  |  |  |
