# Supplementary material for: Selection and identification of a novel ssDNA aptamer targeting human skeletal muscle
Source: Bioact Mater. 2022 May 27;20:166–78. doi: 10.1016/j.bioactmat.2022.05.016 (PMC9157180; doi:10.1016/j.bioactmat.2022.05.016)
Supplement: Multimedia component 2 [file mmc2.docx]

| Number | Sequence | Length |
| --- | --- | --- |
| HSM01 | CCGGACAAAACTTCAGTTTTTATTTCCAGATCCTGGGCATTG | 42 |
| HSM02 | GCCTGCAAATATTTCCGAATGGGATTATTAGGATTCCCGTTC | 42 |
| HSM03 | AGACGAAACTTATTGTTCTCGAAGACCTTCTTTAGCTGTTCT | 42 |
| HSM04 | CCGGCCAGATAATTGTCTCCAAGGATTATAAGGTTCTCGTTC | 42 |
| HSM05 | ACCGAATTCGGACTCCCTGGGAATGAATTCGCCGACGGTACA | 42 |
| HSM06 | CCAGCTGAAAAAAATTCAAAGAATTTTTACGTTCGGGTTTGT | 42 |

**Table 2 Top Six Ranked Aptamers Through Cell-SELEX**
