# Supplementary material for: Selection and identification of a novel ssDNA aptamer targeting human skeletal muscle
Source: Bioact Mater. 2022 May 27;20:166–78. doi: 10.1016/j.bioactmat.2022.05.016 (PMC9157180; doi:10.1016/j.bioactmat.2022.05.016)
Supplement: Multimedia component 3 [file mmc3.docx]

**Table 3 Top Ranked MS Detected Protein**

| Database number | HSM01 | LIB | Protein |
| --- | --- | --- | --- |
| P02751\|FINC_HUMAN | 6400000 | 29500 | Fibronectin |
| P12111\|CO6A3_HUMAN | 76100 | 6980 | Collagen |
| P21333\|FLNA_HUMAN | 22500 | 3740 | Filament protein actin |
| Q15149\|PLEC_HUMAN | 23100 | 1530 | Filamin |
| Q14315\|FLNC_HUMAN | 17200 | 2920 | Filamin |
| Q9Y490\|TLN1_HUMAN | 11700 | 1520 | Talin |
