# Supplementary material for: Selection and identification of a novel ssDNA aptamer targeting human skeletal muscle
Source: Bioact Mater. 2022 May 27;20:166–78. doi: 10.1016/j.bioactmat.2022.05.016 (PMC9157180; doi:10.1016/j.bioactmat.2022.05.016)
Supplement: Multimedia component 4 [file mmc4.docx]

**Table 4** Mass spectroscopy analysis result

| **Protein Accession** | **Description** | **-10lgP** | **Peptides** | **Unique Peptides** | **Coverage(%)** | | **Avg.Mass** |
| --- | --- | --- | --- | --- | --- | --- | --- |
| **P02751\|FINC_HUMAN** | Fibronectin OS=Homo sapiens GN=FN1 PE=1 SV=4 | 390.59 | 112 | 112 | 36 | 262622 | |
| **P04264\|K2C1_HUMAN** | Keratin type II cytoskeletal 1 OS=Homo sapiens GN=KRT1 PE=1 SV=6 | 257.73 | 26 | 15 | 39 | 66039 | |
| **P12111\|CO6A3_HUMAN** | Collagen alpha3(VI) chain OS=Homo sapiens GN=COL6A3 PE=1 SV=5 | 246.66 | 38 | 38 | 12 | 343668 | |
| **P13645\|K1C10_HUMAN** | Keratin type I cytoskeletal 10 OS=Homo sapiens GN=KRT10 PE=1 SV=6 | 241.26 | 22 | 16 | 26 | 58827 | |
| **P35908\|K22E_HUMAN** | Keratin type II cytoskeletal 2 epidermal OS=Homo sapiens GN=KRT2 PE=1 SV=2 | 237.96 | 21 | 12 | 31 | 65433 | |
| **P02533\|K1C14_HUMAN** | Keratin type I cytoskeletal 14 OS=Homo sapiens GN=KRT14 PE=1 SV=4 | 206.65 | 14 | 4 | 35 | 51562 | |
| **P21333\|FLNA_HUMAN** | Filamin-A OS=Homo sapiens GN=FLNA PE=1 SV=4 | 206.4 | 21 | 20 | 11 | 280737 | |
| **P35527\|K1C9_HUMAN** | Keratin type I cytoskeletal 9 OS=Homo sapiens GN=KRT9 PE=1 SV=3 | 204.57 | 12 | 12 | 24 | 62064 | |
| **P13647\|K2C5_HUMAN** | Keratin type II cytoskeletal 5 OS=Homo sapiens GN=KRT5 PE=1 SV=3 | 178.26 | 14 | 2 | 20 | 62378 | |
| **P48668\|K2C6C_HUMAN** | Keratin type II cytoskeletal 6C OS=Homo sapiens GN=KRT6C PE=1 SV=3 | 171.47 | 14 | 2 | 26 | 60025 | |
| **Q15149\|PLEC_HUMAN** | Plectin OS=Homo sapiens GN=PLEC PE=1 SV=3 | 162.03 | 18 | 18 | 4 | 531796 | |
| **Q14315\|FLNC_HUMAN** | Filamin-C OS=Homo sapiens GN=FLNC PE=1 SV=3 | 160.64 | 12 | 11 | 6 | 291020 | |
| **P02768\|ALBU_HUMAN** | Serum albumin OS=Homo sapiens GN=ALB PE=1 SV=2 | 157.28 | 6 | 2 | 9 | 69367 | |
| **P08779\|K1C16_HUMAN** | Keratin type I cytoskeletal 16 OS=Homo sapiens GN=KRT16 PE=1 SV=4 | 147.52 | 12 | 5 | 27 | 51268 | |
| **Q9Y490\|TLN1_HUMAN** | Talin-1 OS=Homo sapiens GN=TLN1 PE=1 SV=3 | 141.22 | 12 | 12 | 5 | 269765 | |
| **Q9NZM1\|MYOF_HUMAN** | Myoferlin OS=Homo sapiens GN=MYOF PE=1 SV=1 | 134.86 | 9 | 9 | 5 | 234706 | |
| **P35579\|MYH9_HUMAN** | Myosin-9 OS=Homo sapiens GN=MYH9 PE=1 SV=4 | 133.05 | 8 | 8 | 5 | 226530 | |
| **Q7Z794\|K2C1B_HUMAN** | Keratin type II cytoskeletal 1b OS=Homo sapiens GN=KRT77 PE=2 SV=3 | 114.25 | 5 | 2 | 7 | 61901 | |
| **P60709\|ACTB_HUMAN** | Actin cytoplasmic 1 OS=Homo sapiens GN=ACTB PE=1 SV=1 | 104.49 | 5 | 4 | 15 | 41737 | |
| **P63261\|ACTG_HUMAN** | Actin cytoplasmic 2 OS=Homo sapiens GN=ACTG1 PE=1 SV=1 | 104.49 | 5 | 4 | 15 | 41793 | |
| **O75369\|FLNB_HUMAN** | Filamin-B OS=Homo sapiens GN=FLNB PE=1 SV=2 | 92.01 | 2 | 1 | 1 | 278162 | |
