# Supplementary material for: Selection and identification of a novel ssDNA aptamer targeting human skeletal muscle
Source: Bioact Mater. 2022 May 27;20:166–78. doi: 10.1016/j.bioactmat.2022.05.016 (PMC9157180; doi:10.1016/j.bioactmat.2022.05.016)
Supplement: Multimedia component 5 [file mmc5.docx]

**Table 5** The ssDNA aptamer 3D structure prediction result

| **Name** | **Sequence** | **Predicted Structure** |
| --- | --- | --- |
| HSM01 | CCGGACAAAACTTCAGTTTTTATTTCCAGATCCTGGGCATTG | 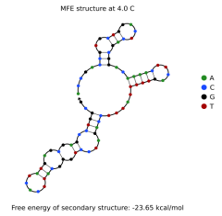 |
| HSM02 | GCCTGCAAATATTTCCGAATGGGATTATTAGGATTCCCGTTC | 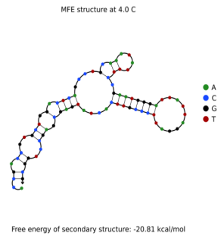 |
| HSM03 | AGACGAAACTTATTGTTCTCGAAGACCTTCTTTAGCTGTTCT | 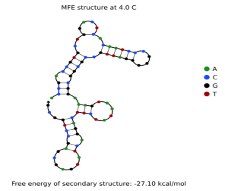 |
| HSM04 | CCGGCCAGATAATTGTCTCCAAGGATTATAAGGTTCTCGTTC | 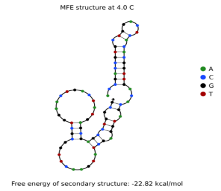 |
| HSM05 | ACCGAATTCGGACTCCCTGGGAATGAATTCGCCGACGGTACA | 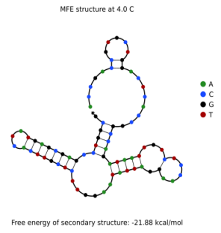 |
| HSM06 | CCAGCTGAAAAAAATTCAAAGAATTTTTACGTTCGGGTTTGT | 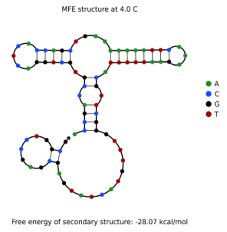 |
